# Reviews and Book Notices

**Published:** 1883-02

**Authors:** 


### Reviews and Book Notices.

ARTICLE VII.—MICROSCOPICAL MORPHOLOGY OF THE ANIMAL BODY IN HEALTH AND DISEASE. By C. HEITZMANN, M.D. Late Lecturer on Morbid Anatomy, at the University in Vienna. With 380 Original Engravings. 8vo., cloth, pp. 849. New York: J. H. Vail & Co. 1883.

This is a great work whose merits cannot be ascertained by a mere perusal, and yet one could not extol it as a valuable *text-book*. To a great many it will remain but a stupendous contribution to the science of histology, which is in its infancy though a robust child. A large part of the book was written ten years ago, and consists of a translation of previous papers by the author, too much of it being mere discussion. The effort of Dr. Heitzmann at demolishing the cell doctrine is like that of the socialist to do away with the government. In both cases the narrow mind of man fails to see the compatibility of a foundation with a superstructure.

The cell theory is one of the greatest modern scientific achievements, a resting platform in the stairs of knowledge, and the merit of Dr. Heitzmann's researches is this, that he has shown that the *cells* have a regular ground-work which former investigation had not seen. Another advantage of the book, most of the illustrations are original, as they should be in such cases, though a great many are diagramatic, which is hardly excusable in works on microscopy.

In the preface, the author refers to the support he received from a wealthy friend. This is a detriment, for we live in an age when practical scientific books are in such demand as to pay for the publication; except, perhaps, when a writer tries to make a text-book of an aggregation of previous pamphlets. The 12 pages devoted to methods are very practical, and one wishes that a longer space had been given to the subject. The chapter on the General Properties of Living Matter, is extremely crude compared with the treatment of the same subject by Hadley, in his "Anatomy of the Invertebrate Animals," or Spencer, in his "Biology." The net-work of which cells ultimately consist is described in chapter iii. as observed by the author in examining amœbæ with a 1000 power. It is well to state here that other observers have failed to discover such structure, but in this as in other cases analogy suggested the discovery and it was not made through the help of the best lenses, though it is doubtless a most important one.

The substitution of tissue *elements* for *cells* is only misleading, and the old term should be preserved for many obvious reasons.

The communication on "The Structure and Origin of Colored Blood-Corpuscles," by Louis Elsberg, forms one of the most valuable and interesting chapters. Dr. Heitzmann's work is well adapted for those students who are particularly fond of investigations rather than memorizers of known facts, and several views of the author will certainly stimulate microscopists to more accurate observations. Besides, a careful elimination of a good deal of useless material, would make a valuable text-book of what seems incongruous matters in this first edition. The printing as well as the engraving leave no desideratum.

ARTICLE VIII.—A MANUAL OF OBSTETRICS. By A. F. A. KING, M.D., Professor of Obstetrics and Diseases of Women and Children in the Medical Department of the Columbian University, Washington, D. C., and in the University of Vermont, etc., etc. With 58 Illustrations. 12mo, cloth, pp. 325. Philadelphia: Henry C. Lea's Son & Co. 1882.

Of all the manuals issued of late in the various departments of medicine, none compare with this. It is the clearest, neatest, most complete and most interesting work on the subject of which it treats, and it seems that Prof. King has succeeded in conveying in its chapters the wonderful interest which attached to his lectures when delivered before his attentive students.

Although the author has often published valuable contributions to the science of obstetrics, his greatest glory is the magic affection with which all his students remember him, and his aim in dedicating his book to them has been to make a book which answered their wants in every particular, and in that he has entirely succeeded.

This volume is much more thorough than a first glance would reveal, for it comprises even obstetric jurisprudence, while, by a system of condensation peculiar to Prof. King, one wonders at the number of facts expressed in a paragraph, and at the numerous details of treatment conveyed in half a page. The illustrations are remarkable for their accuracy, and this volume is issued in the best style of the well-known publishers.

Although best intended for students, this book is also very useful in the library as a reference work, for it contains all the essentials of the larger works on the subject, besides many original suggestions. And the treatment is everywhere exhaustive. This volume also possesses the rare merit of slipping inside an overcoat pocket, so that it can easily be taken along to the bedside, where accoucheurs sometimes have to pass such weary hours if they have no reading matter at hand.

H. D. V.

ARTICLE IX.—THE PHARMACOPŒIA OF THE UNITED STATES OF AMERICA. Sixth Decennial Revision. By authority of the National Convention for Revising the Pharmacopœia, held at Washington, A. D. 1880. 8vo, pp. 488. New York: William Wood & Co. 1882.

On the whole, this edition will be very satisfactory to the greatest number of readers. The introduction of abstracts of various drugs forms a convenient mode of administering them. So the trituritions with sugar of milk.

The preparations of opium in this new edition are stronger than in the former, and will be a source of misunderstandings between prescribers and druggists until both become accustomed to the change. Most of the ingredients of compounds are taken as so many parts in a hundred ; in some both the metric and the old systems are used. A great many fl. extr. syrups and trs.

have been added, but comparatively few new remedies. Every drug appears in alphabetical order, with a prominent name, and a great deal of blanks. In fact, it is probable that the high price of the book, in which these blank spaces occupy such prominence, will prevent its general use by prescribing physicians. This book could have been issued in a more economical manner. However, an objection of a totally different character will be made by many to the small type used in describing many substances. More plain type and less *leading* would have made a prettier and more useful volume.

As it is, neveertheless, it forms the best reference book in prescription writing. It is indispensable to druggists, and it is the materia medica recognized by the law so that it is indispensable to most physicians.

It is issued in a variety of styles, to answer the wants of druggists especially. H. D. V.

ARTICLE X. — ANATOMICAL TECHNOLOGY AS APPLIED TO THE DOMESTIC CAT. An Introduction to Human, Veterinary, and Comparative Anatomy. By Burt G. Wilder, B.S., M.D., and Simon H. Gage, B.S. (Both of Cornell University). 8vo., cloth, pp. 575. New York and Chicago: A. S. Barnes & Co., 1882.

A timely and most valuable work on anatomy. This is certainly the most practical book on the subject, and it will prove of the greatest benefit to those who pursue their study alone, or dissect without the help of a demonstrator. The large number of Latin anatomical terms used, while easily acquired by a short study of the language, impart to the subject a sort of international homogeneity which cannot be too highly commended. Although the greatest attention is given to the anatomy of the cat, this manual is the best work available on the dissection of any mammal, and must form the best of introductions to human anatomy. However, as medical students have but little time to give to dissection while pursuing the study of medicine, and as the anatomy of man is the only one that they can ever master, they should not make the mistake of entering the profession with nothing but a smattering of the anatomy of the cat, and, therefore, we would not recommend the introduction of this work in the medical colleges of this country. But, for those physicians who dissect whenever an opportunity offers, who are interested in comparative anatomy, or practice vivisection, this is the best hand-book. With naturalists at large, a rapidly growing class in this age, a want long felt has just been filled, and it is probable that it could never have been filled in a better manner, except, perhaps, for the wood-cuts.

ARTICLE XI. — PATHOLOGICAL ANATOMY. Pathology and Physical Diagnosis. A Series of Clinical Reports, Comprising the Principal Diseases of the Human Body, Systematically Arranged in One Hundred Full page Illustrations and One Hundred Pages Text. By I. A. JEANCON, M.D. Progress Publishing Company, Cincinnati. Twenty-five Parts. Price $1.00 per Part.

We have before us, Section One of this promising publication. It treats of diseases of the cerebro-spinal axis and its membranes, beginning with sub-arachnoidal meningitis of the cortex and surface of the brain (comatose form).

Table One illustrates beautifully, by eight chromo-lithographic figures, three well reported cases of cerebral arachnitis.

Table Two illustrates, in an equally artistic manner, by four figures, the post-mortem appearance of a case of hemiplegia, with excessive pain in the limbs and epileptiform spasms; two cancerous tumors of the dura mater; gelatiniform softening of the cerebral substance.

Table Three illustrates, by six admirable figures, tumors of the falx cerebri, and the accompanying text gives the histories of two cases from which the specimens were obtained.

Table Four contains four figures, that represent, in an equally creditable manner, acute meningitis of the cerebrum and cerebellum, and the text gives complete histories of two cases from which the illustrations were obtained.

We can emphatically commend this work to the profession.

D. R. B.

ARTICLE XII.—ESSENTIALS OF VACCINATION; A Compilation of Facts Relating to Vaccine Inoculation and its Influence in the Prevention of Small-pox. By W. A. Hardaway, M.D., Formerly one of the Vaccine Physicians to the City of St. Louis, etc. 12mo., cloth, pp. 146, $1.00. Chicago: Jansen, McClurg & Co., 1882.

Of the various valuable contributions made to the subject during a few years this seems the best and most practical. It comprises chapters on the history of vaccination, vaccina in animals and in man, complications, etc., revaccination, merits of different kinds of virus, etc. It is terse and thorough, not encumbered by technicalities, and it is issued in very good style. While of great value to the profession, this monograph is also very well adapted for the people at large, especially in these days of anti-vaccination crazes.

---

COOK COUNTY HOSPITAL.—Dr. Jas. M. Hutchinson is giving an excellent surgical clinic every Tuesday at the hospital. His dissertations show that he has within him the elements of a successful clinician.

DR. F. Henrotin is giving an acceptable medical clinic every Tuesday.

Dr. A. J. Baxter is conducting a surgical clinic every Friday.

There is no medical clinic on Friday.

It is a matter of great regret that so many of the present medical staff of the hospital have no capacity for and no experience in medical teaching, and that, in consequence, the abundant material is not used as it should be to encourage the progress of medical education in Chicago. Chicago is the great medical center, as it is the great commercial center, of the Northwest, and the county commissioners should see to it that competent teachers, as well as capable practitioners, constitute the Hospital staff.

